# Supplementary material for: ENT3C: an entropy-based similarity measure for Hi-C and micro-C derived contact matrices
Source: NAR Genom Bioinform. 2024 Jul 2;6(3):lqae076. doi: 10.1093/nargab/lqae076 (PMC11217677; doi:10.1093/nargab/lqae076)
Supplement: lqae076_Supplemental_File [file lqae076_supplemental_file.pdf]

Supplementary Material
ENT3C: an entropy-based similarity measure for Hi-C and micro-C derived contact matrices

Xenia Lainscsek and Leila Taher

Institute of Biomedical Informatics, Graz University of Technology, Graz, Austria

Supplementary Figures

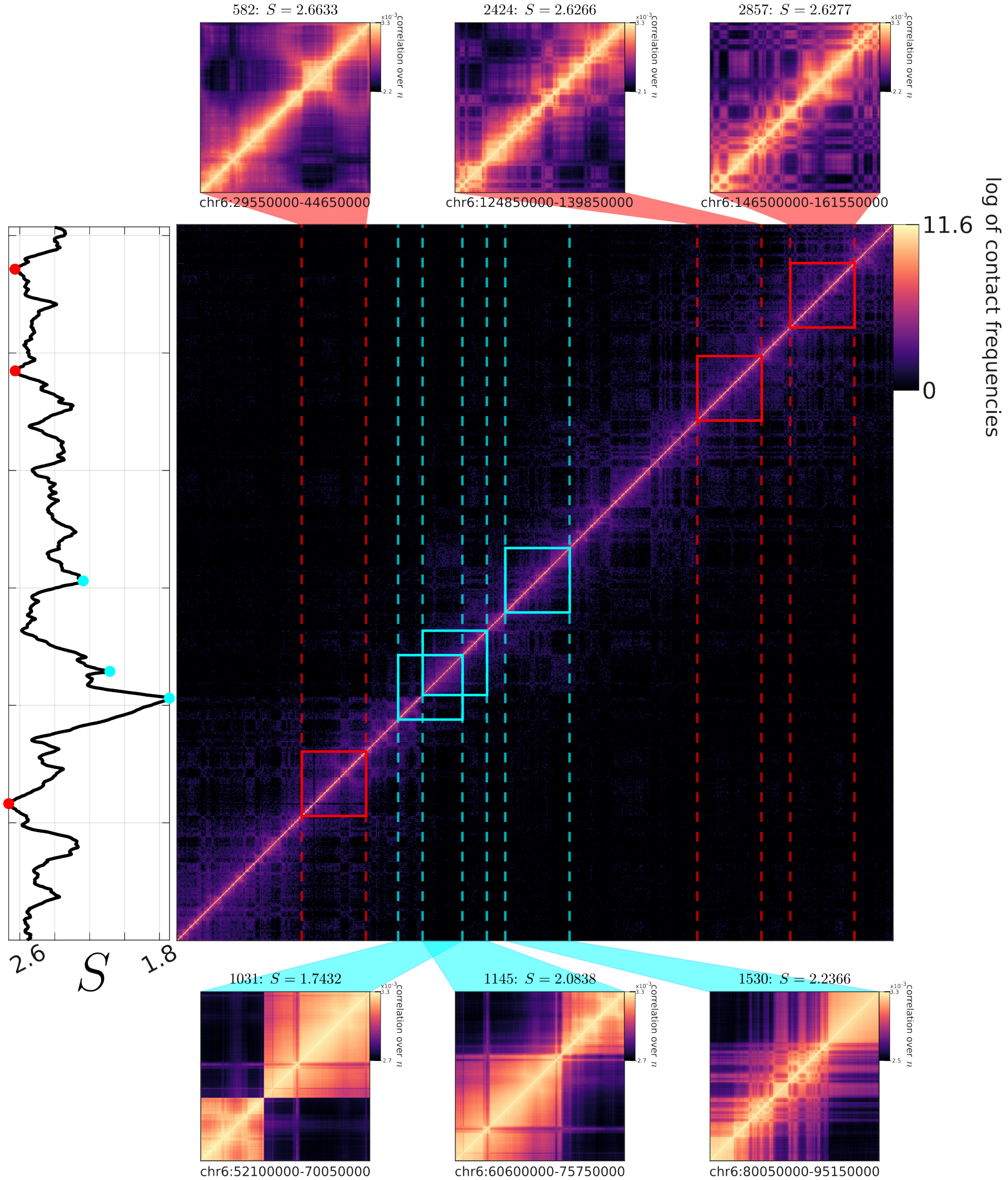

Figure S1. Entropy curve S of chromosome 6 for the first biological replicate of the HFFc6 cell line at 50 kb binning resolution. The von Neumann entropy summarizes the frequency with which chromatin states change (chromatin state dynamics) in a given genomic region. Compared to high entropy values (shown by red dots on the curve), low entropy values (cyan dots) are reflected in less complex patterns in the corresponding submatrices. ENT3C parameters were set to: $n = 300$, $\phi = 5$, and $\Phi_{max} = \infty$.

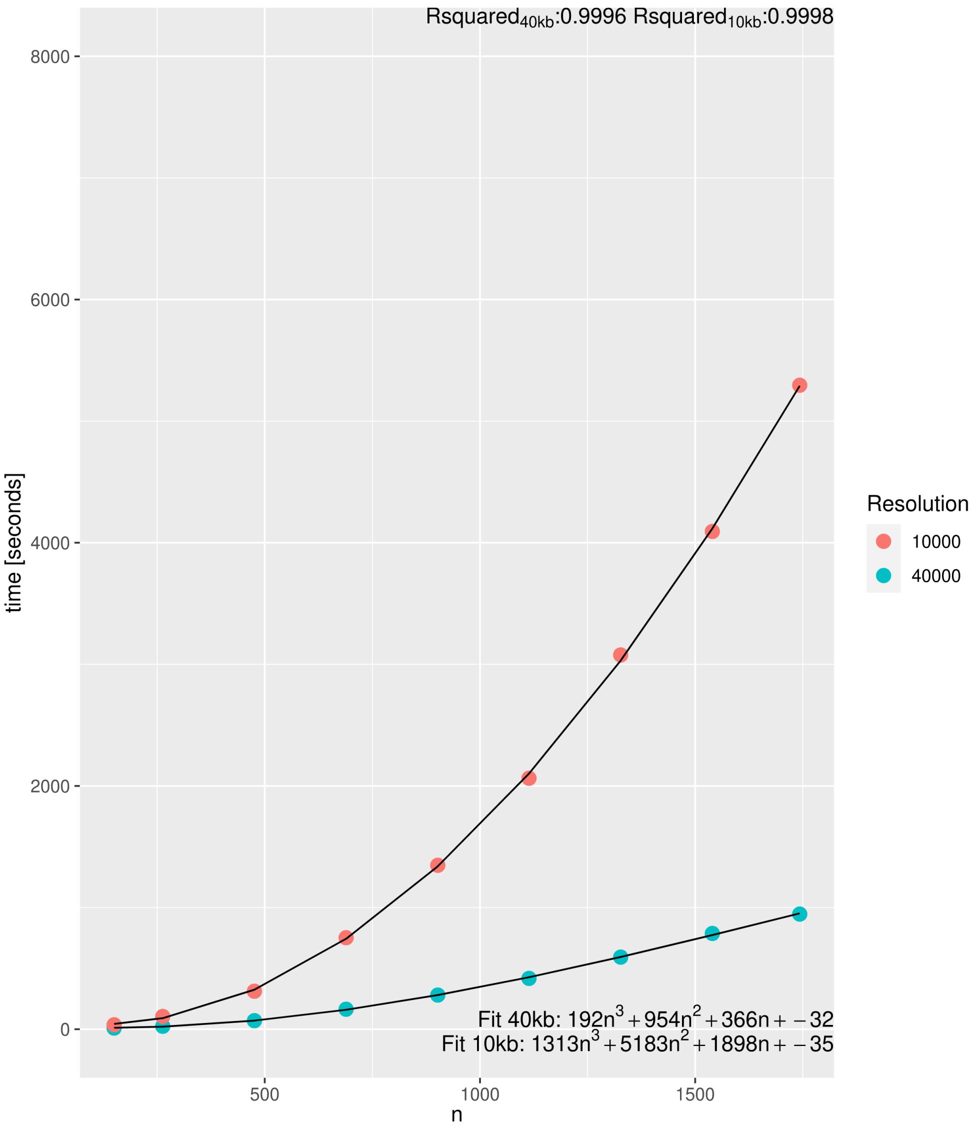

Figure S2. ENT3C has $\mathcal{O}(\Phi{\cdot}n^3)$ time complexity. The two main factors contributing to ENT3C's time complexity are the transformation of the submatrices to Pearson matrices and the subsequent eigenvalue decomposition, which are both approximately $\mathcal{O}(n^3)$. The number of times these computations need to be performed depends on the size of the input matrix N and size of the submatrix n and are related as: $\Phi = 1 + \lfloor \frac{N-n}{\varphi} \rfloor$. The time it takes for ENT3C to analyze chromosome 1 binned at 10 kb and 40 kb can be approximated by a third-degree polynomial in n. This analysis was run with MATLAB version 9.14.0.2337262 (R2023a) Update 5 on an AMD© Ryzen 9 3900×12-core processor×24 running Ubuntu 20.04.6 LTS.

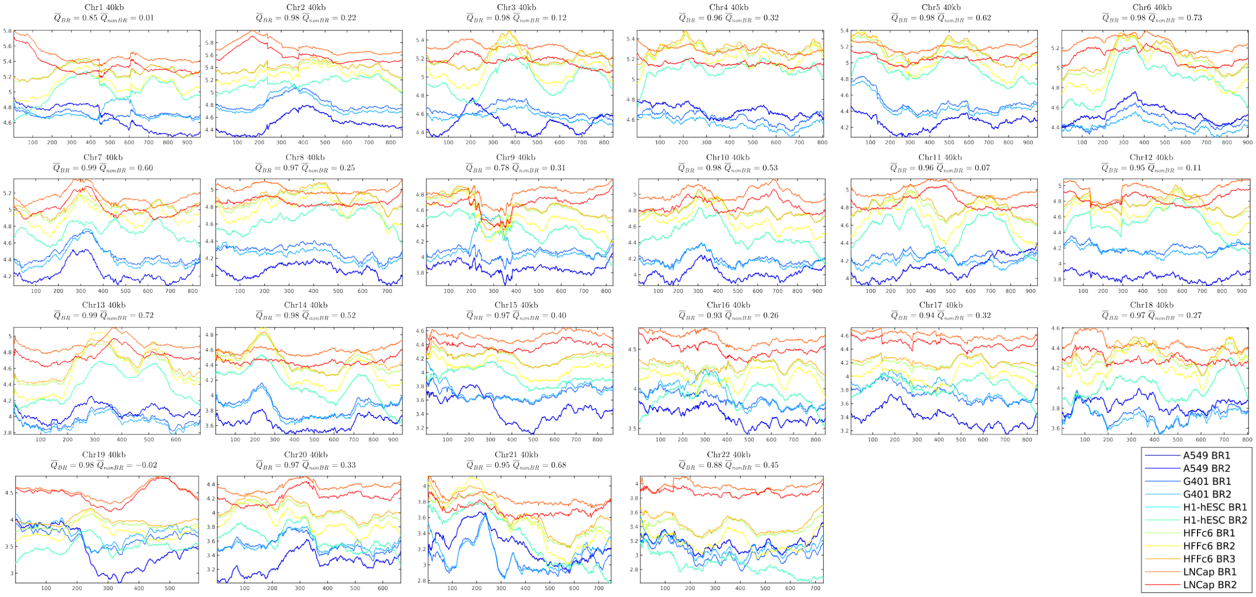

Figure S3. ENT3C uses the Pearson correlation of entropy signals S to define contact matrix similarity. **S** is shown for 40 kb-binned contact matrices from pairs files downsampled to 30 million interactions in various cell lines. Titles indicate ENT3C similarities Q (Methods) between contact matrices derived from biological replicates of the same cell line (BR) and non-replicates derived from different cell lines (NR). BRs are represented in similar color schemes. ENT3C parameters were set to: $c = 7$, $\varphi = 1$, and $\Phi_{max} = 1000$.

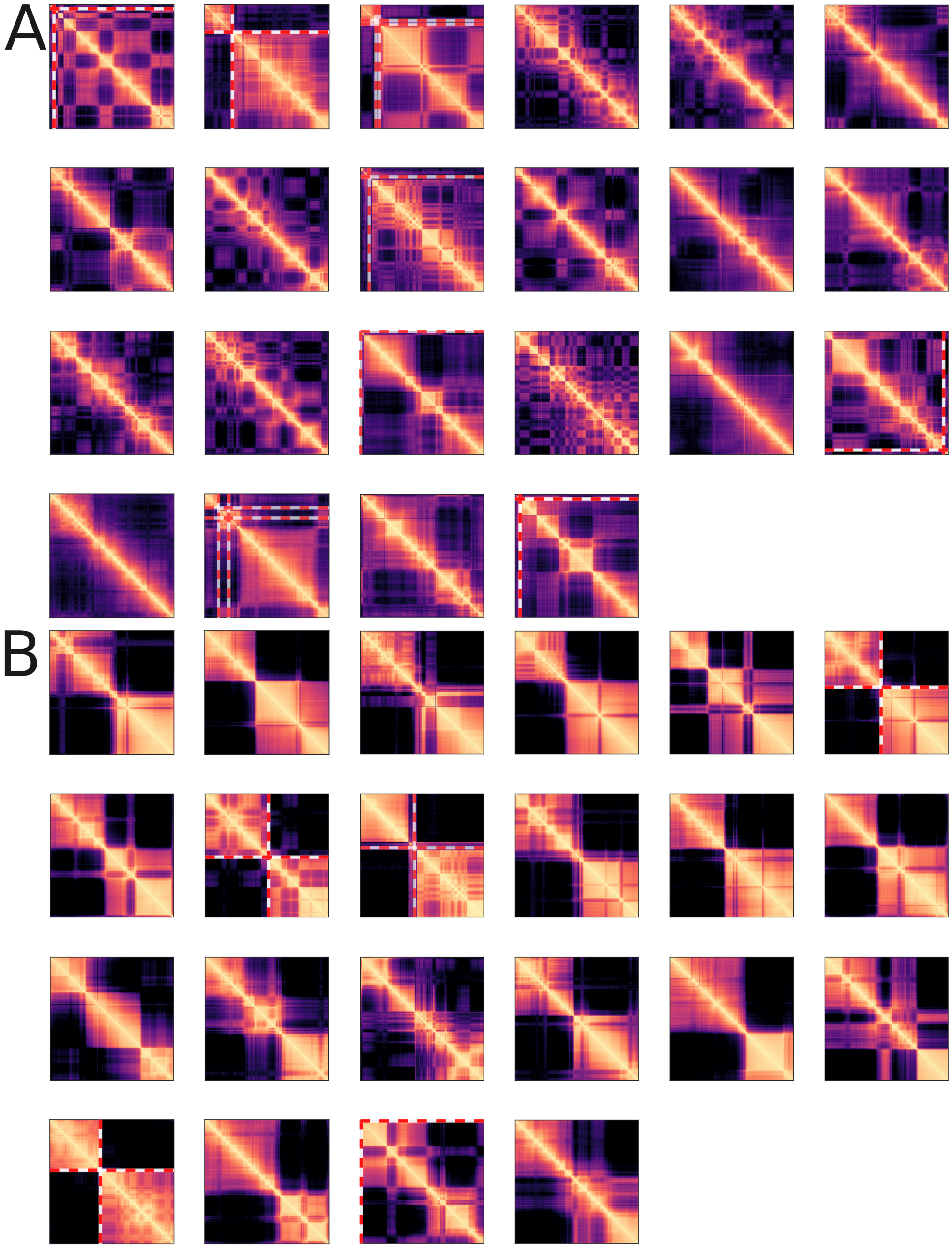

Figure S4. Highest and lowest entropy values correspond to higher and lower pattern complexity, respectively. Submatrices corresponding to maximum (**A**) and minimum (**B**) entropy values for 40 kb-binned HFFc6 contact matrices (pooled biological replicates) of each chromosome. ENT3C parameters were set to: $n = 300$, $\varphi = 10$, and $\Phi_{\max} = \infty$. White and red stripes indicate centromeric regions.

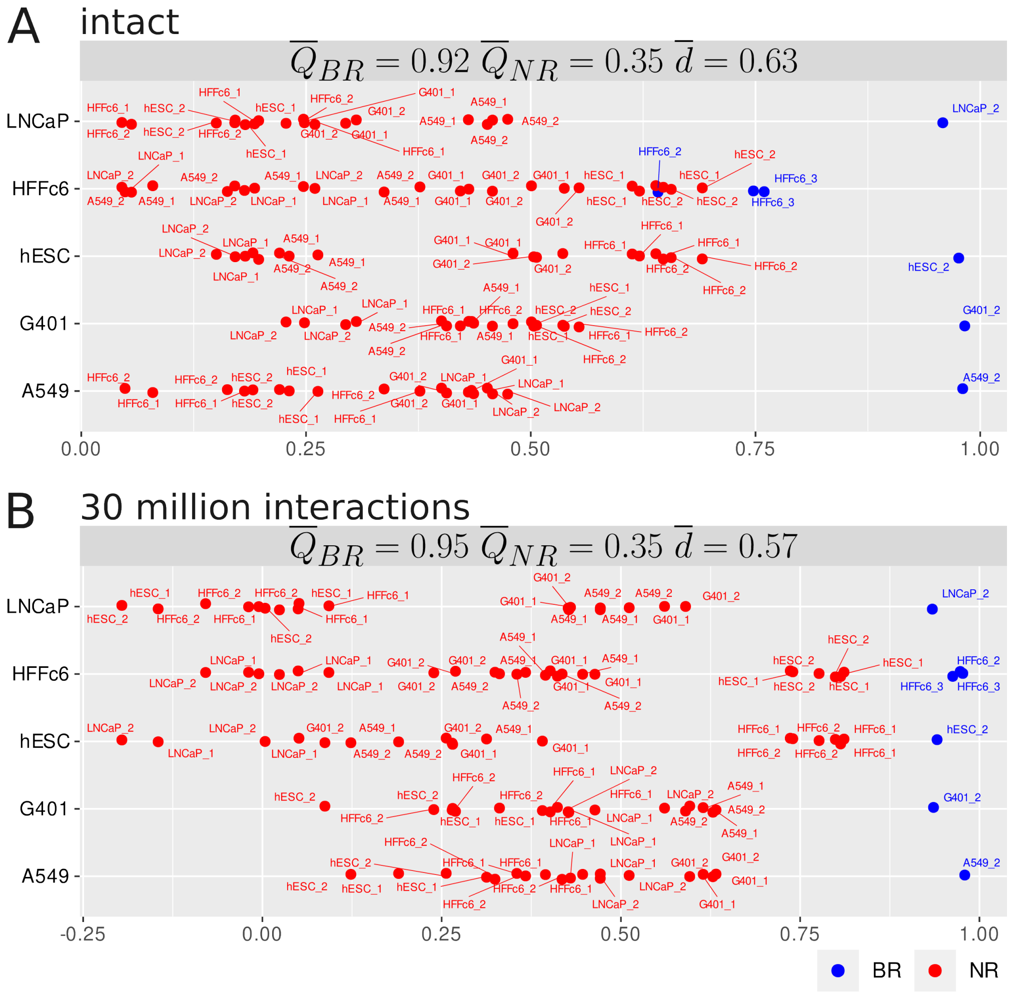

Figure S5. ENT3C distinguishes biological replicate (BR) contact matrices from non-replicate (NR) contact matrices. ENT3C similarity scores between BR and NR pairs of 40 kb-binned **(A)** intact contact matrices and **(B)** contact matrices generated from pairs files downsampled to contain 30 million interactions. Each dot represents the similarity score averages across the autosomes. ENT3C average similarity scores and separating margins across cell lines ($\overline{Q}_{BR}$, $\overline{Q}_{NR}$ and $\overline{d}$) are indicated in the titles (as in Figure 2; Methods). ENT3C parameters were set to: $n = 300$, $\varphi = 10$, and $\Phi_{\max} = \infty$.

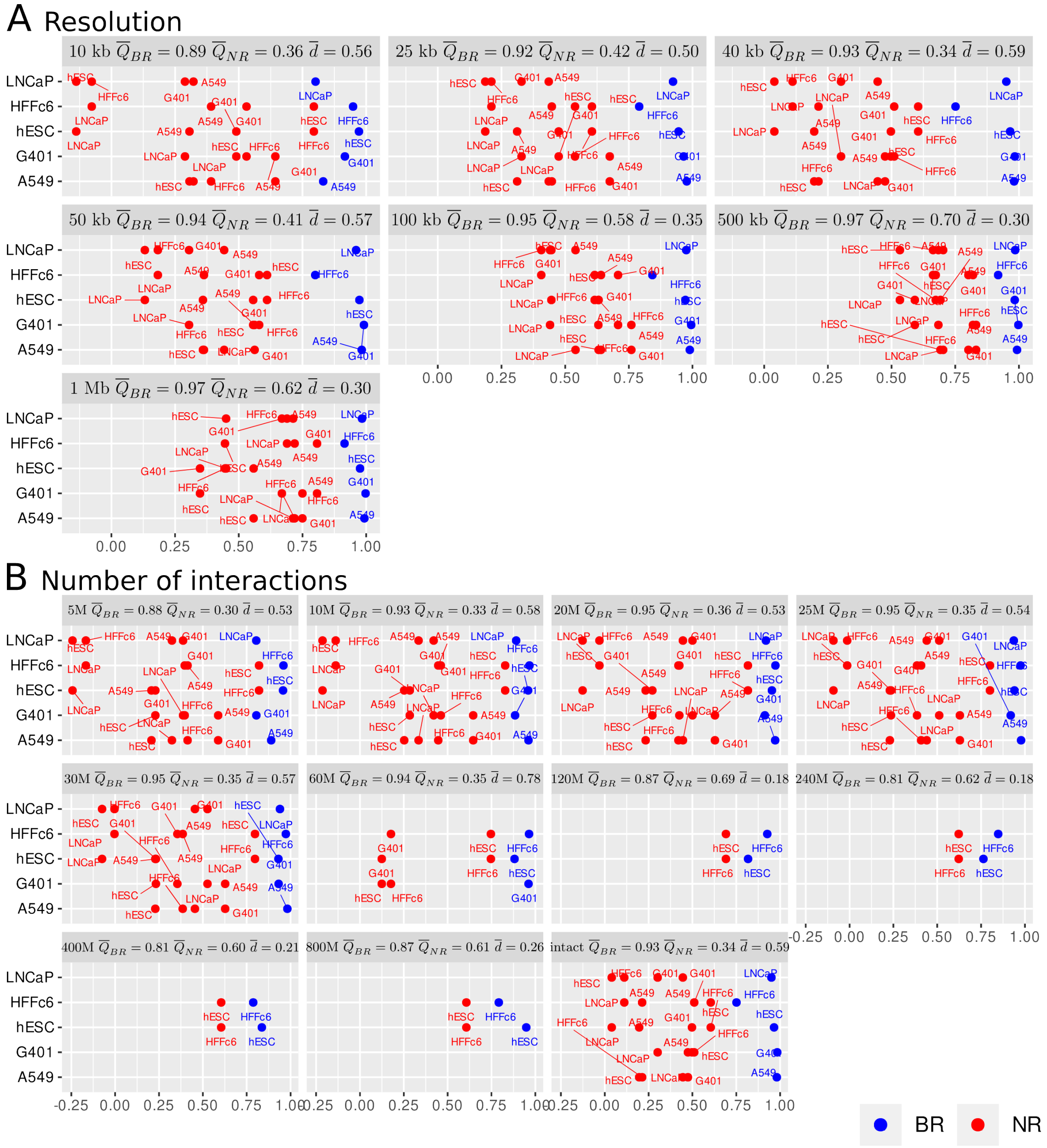

Figure S6. ENT3C is insensitive to binning resolution and sequencing depth. Each dot represents ENT3C average similarity scores Q^{i}_{BR} and $Q^{i,j}_{NR}$ (as in Figure 2; Methods) between pairs of **(A)** intact contact matrices binned at 10, 25, 40, 50, 100, 500 and 1000 kb resolutions and **(B)** 40 kb contact matrices generated from pairs files downsampled to 5, 10, 20, 25, 30, 60, 120, 240, 400, 800 million interactions (the last panel indicates intact contact matrices). ENT3C average similarity scores and separating margins across cell lines ($\overline{Q}_{BR}$, $\overline{Q}_{NR}$ and $\overline{d}$) are indicated in the titles (Methods). ENT3C parameters were set to: $c = 7$, $\varphi = 1$, and $\Phi_{\max} = 1000$.

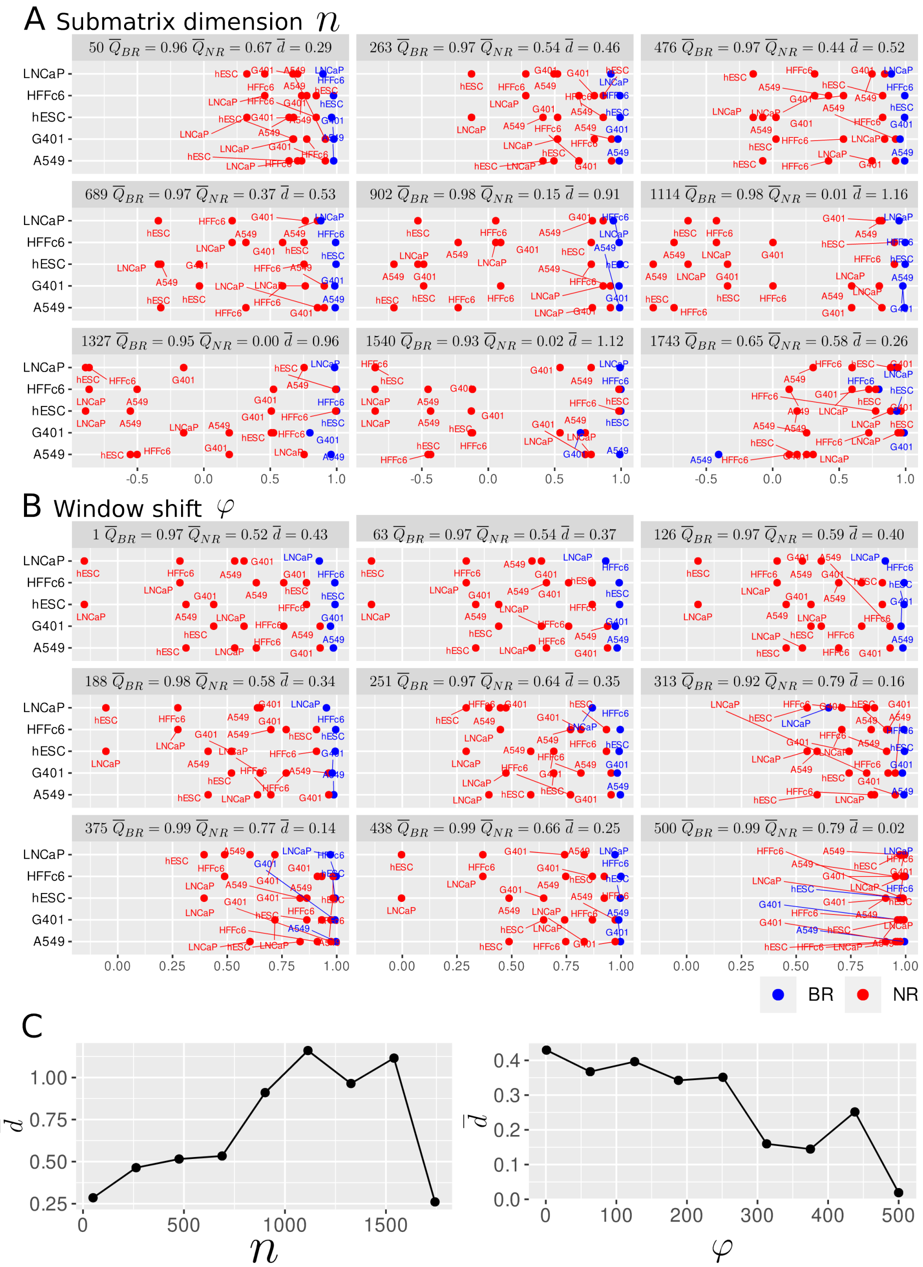

Figure S7. ENT3C is stable to parameter choice. Each dot represents ENT3C similarity scores of chromosome 14 Q^{i}_{BR} and $Q^{i,j}_{NR}$ averaged over replicates average similarity scores Q^{i}_{BR} and $Q^{i,j}_{NR}$ (see Figure 2; Methods). Contact matrices for chromosome 14 were generated from pairs files downsampled to 30 million interactions and binned at 40 kb. (**A**) ENT3C's window shift $\varphi = 1$ and maximum number of matrices evaluated $\Phi_{\max} = 1000$ were fixed and the submatrix size n was varied between 50 and 1743. (**B**) ENT3C's submatrix dimension $n = 300$ and maximum number of matrices evaluated $\Phi_{\max} = 1000$ were fixed, and the window shift φ was varied between 1 and 500. (**C**) Summary of (A) and (B) as the average separating margins across cell lines $\bar{d}$ over ENT3C parameters n and φ (Methods).

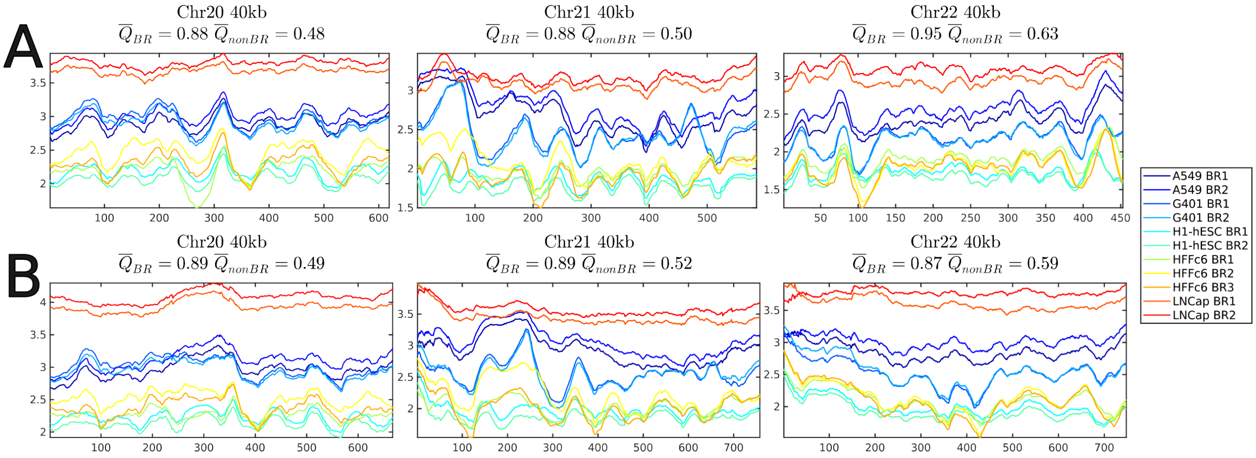

Figure S8. ENT3C displays minor differences in entropy signals when applied to balanced (A) and unbalanced (B) matrices. ENT3C removes empty bins common to the contact matrices being analyzed; additional bins may become empty after balancing. Contact matrices were binned at 40 kb. ENT3C parameters were: set to: $c = 7$, $\varphi = 1$, and $\Phi_{\max} = 1000$.

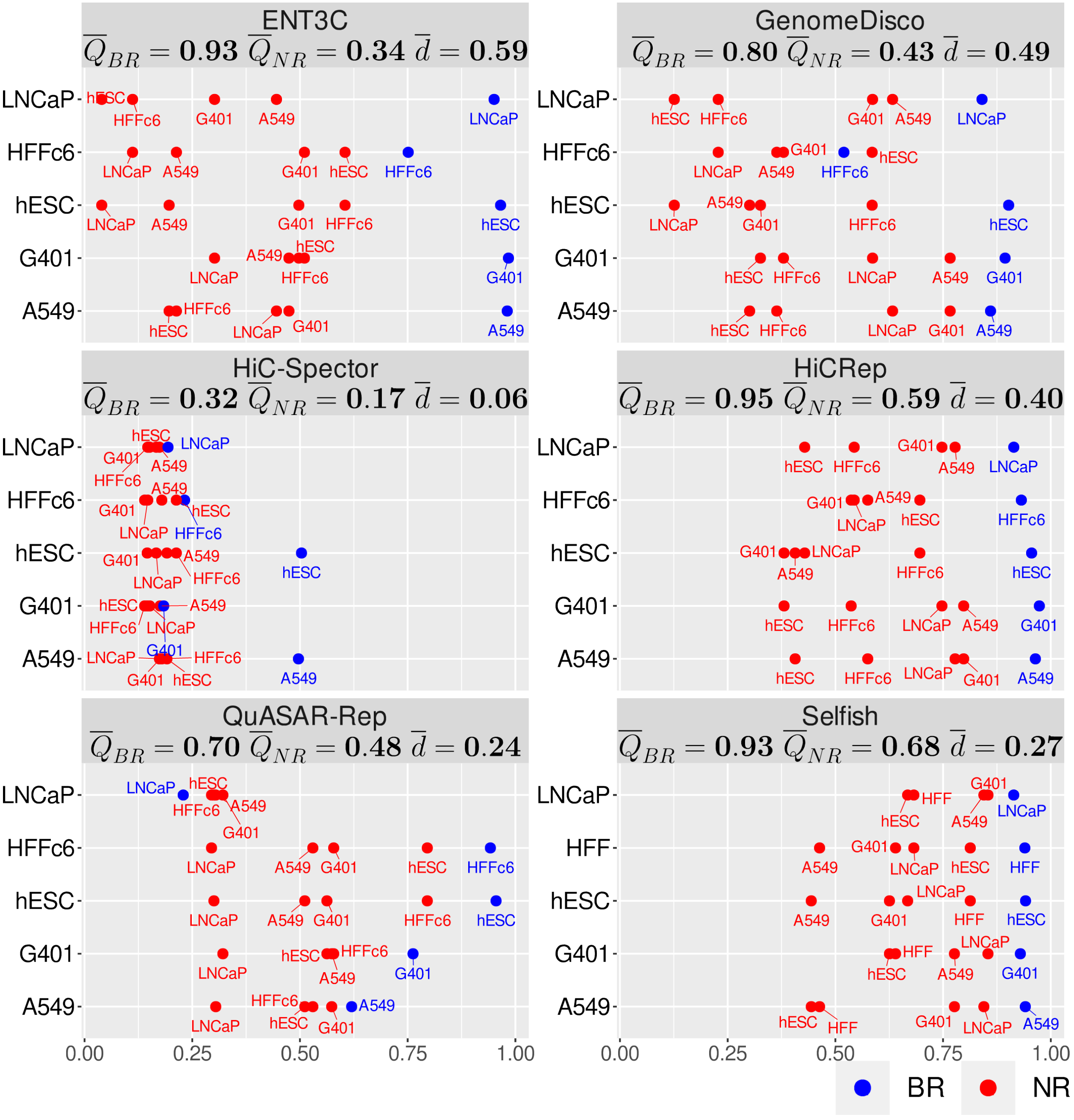

Figure S9. ENT3C competes well with other methods quantifying Hi-C or micro-C contact matrix similarity. Each panel represents a tool (ENT3C, GenomeDISCO, HiC-Spector, HiCRep, QuASAR and Selfish) and each dot represents an average similarity score, either Q_{BR} or Q_{NR} (as in Figure 2-3; Methods). Intact 40 kb-binned contact matrices were used. ENT3C average similarity scores and separating margins across cell lines ($\overline{Q}_{BR}$, $\overline{Q}_{NR}$ and $\overline{d}$) are indicated in the titles (Methods). ENT3C parameters were set to: $c = 7$, $\varphi = 1$, and $\Phi_{\max} = 1000$.

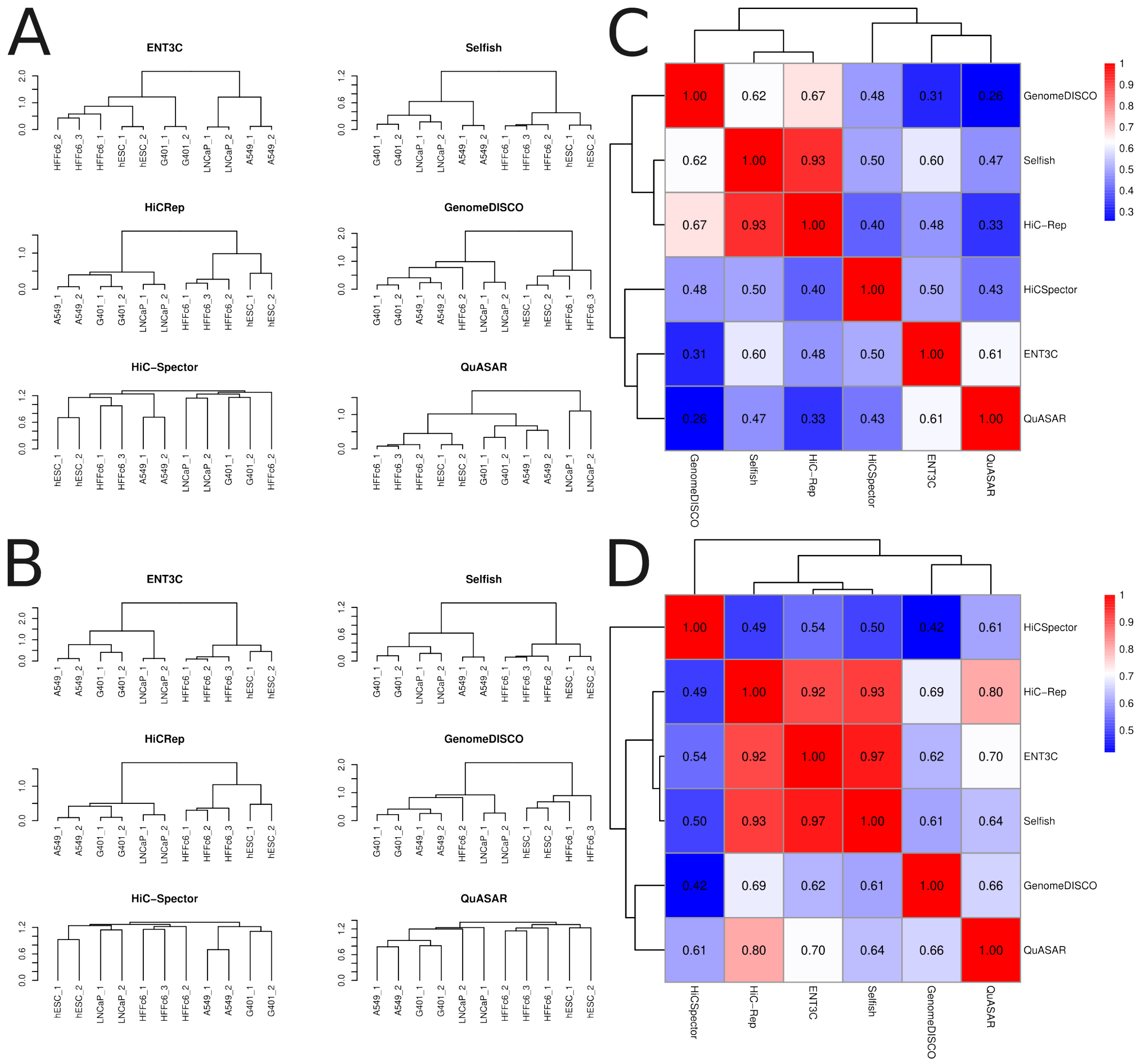

Figure S10. Hierarchical clustering of the samples based on their similarity scores Q shows moderate agreement between different methods. Dendrograms of hierarchical clustering using each method's similarity metric for (**A**) intact and (**B**) downsampled (30×10^6) contact matrices and heatmap of the cophenetic correlation coefficients between the distance matrices obtained from each of the corresponding dendrograms. Agglomerative hierarchical clustering was performed using complete linkage with `R`'s `hclust()` function for each method. Distance was defined as 1 minus the calculated similarity measure. (**C**, **D**) Heatmaps visualizing the correlation coefficient matrices obtained for pairs of all cophenetic distance matrices in (A) and (B) with the `cor.dendlist()` function from R's "dendextend" package.

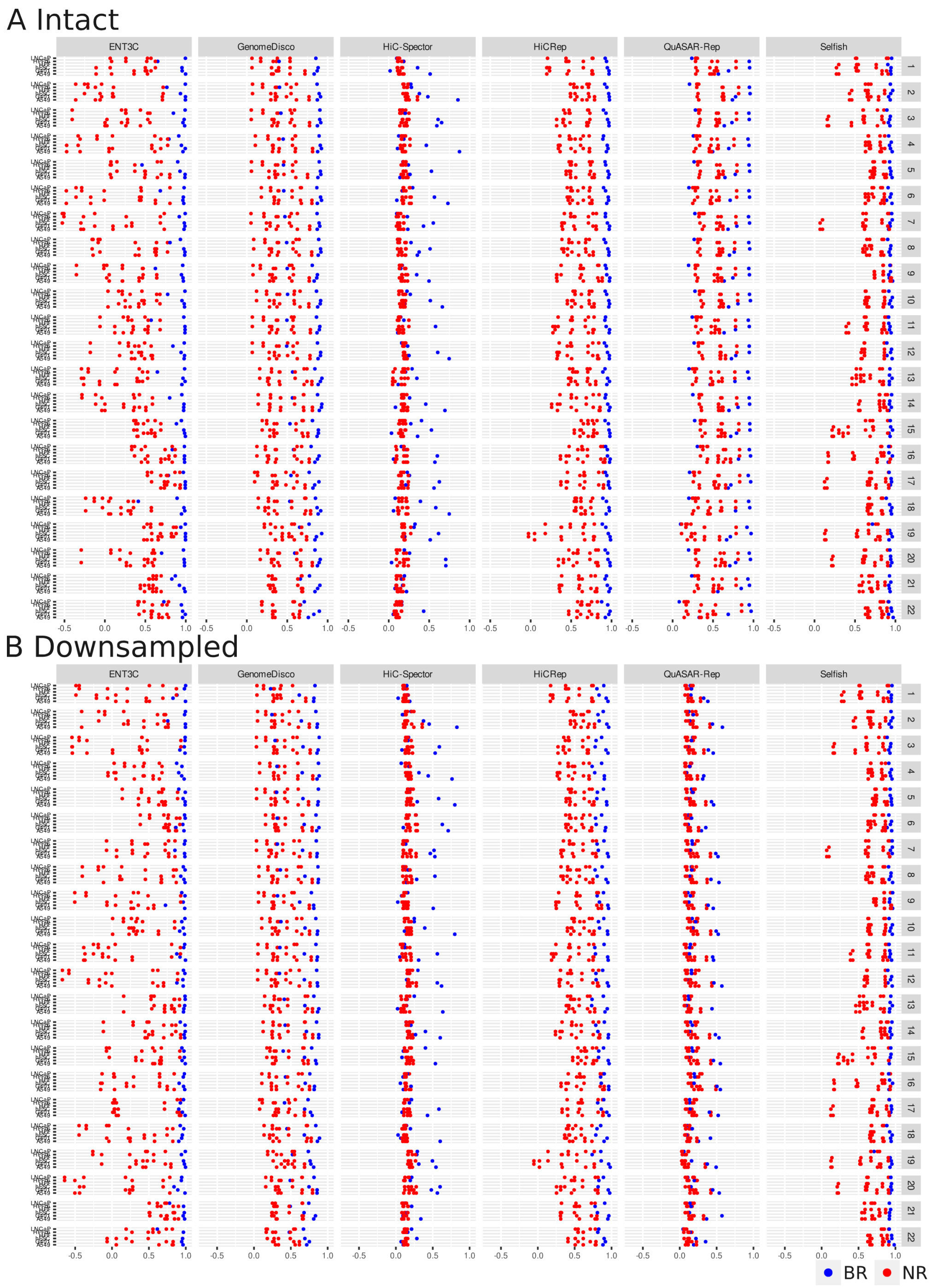

Figure S11. Contact matrix similarity measures often exhibit chromosomal dependency. Each dot represents ENT3C similarity scores averaged over replicates between pairs of **(A)** intact contact matrices and **(B)** contact matrices generated from pairs files downsampled to 30 million interactions. ENT3C parameters were set to: $c = 7$, $\varphi = 1$, and $\Phi_{\max} = 1000$.

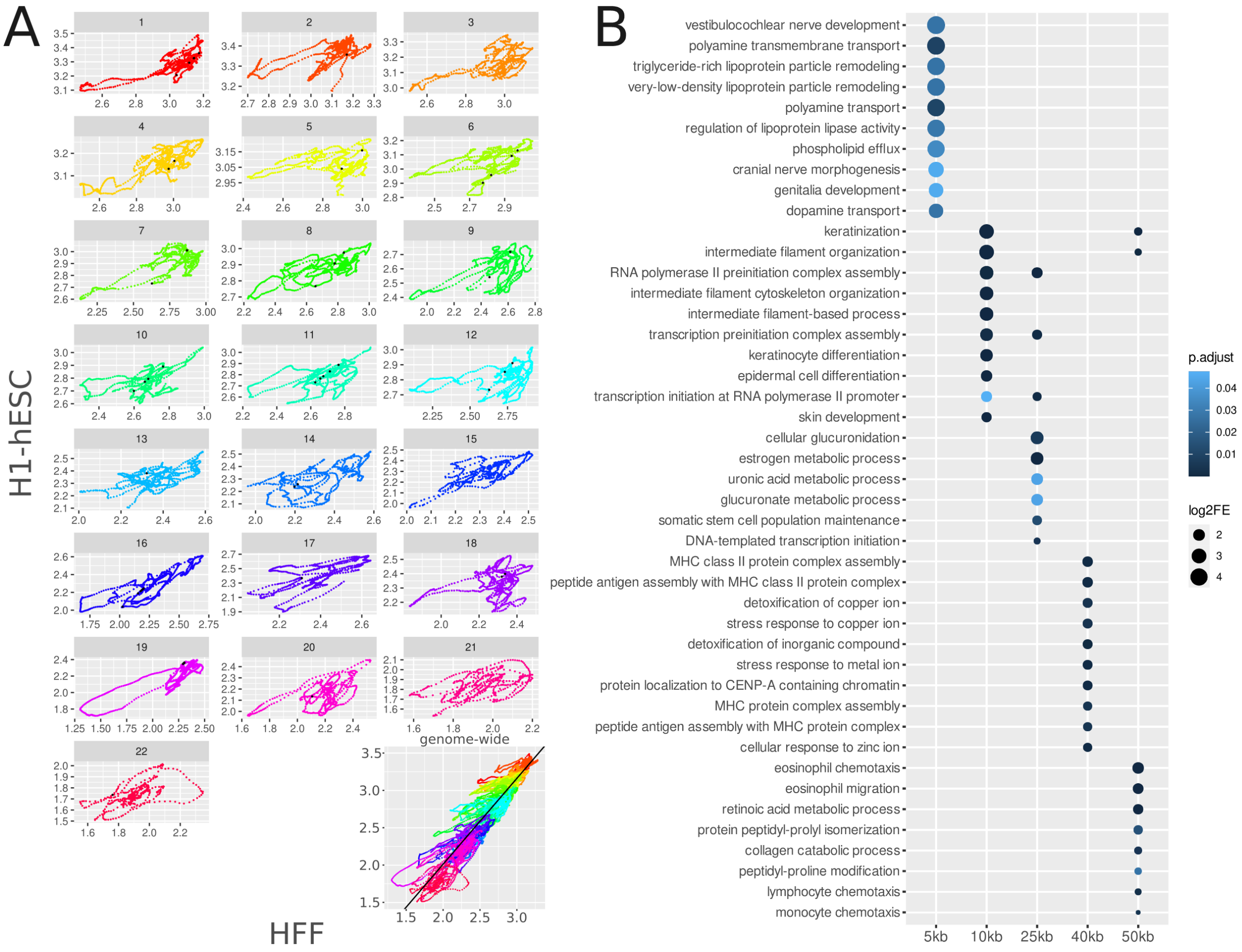

Figure S12. ENT3C's entropy signals can be used for investigating the biological role of similarly complex regions between two cell lines. (**A**) Data points represent the entropy values of HFFc6 (x-axis) and H1-hESC (y-axis) at 40 kb $c = 7$, $\varphi = 1$ and $\Phi_{MAX} = 2000$. The bottom right panel shows the genome-wide data, with the least-squares regression line shown in black. The remaining panels display data from each individual chromosome. Each chromosome is shown in a different color. Black points represent the of data closest (below the 0.3% quantile) to the genome-wide fitted regression line, which were considered regions of most similar complexity. (**B**) Top 10 GO terms arranged by $\log_2$-fold enrichment associated with the genes in the most similar regions at different resolutions (x-axis). For the other resolutions, ENT3C parameters were set to: $c = 150$ for 5 kb, $c = 100$ for 10 kb, $c = 25$ for 25 kb, $c = 7$ for 50 kb, $c = 6$ for 100 kb, $c = 3$ for 500 kb, $c = 2$ for 1 Mb. For all resolutions, $\varphi = 1$ $\Phi_{MAX} = 2000$.

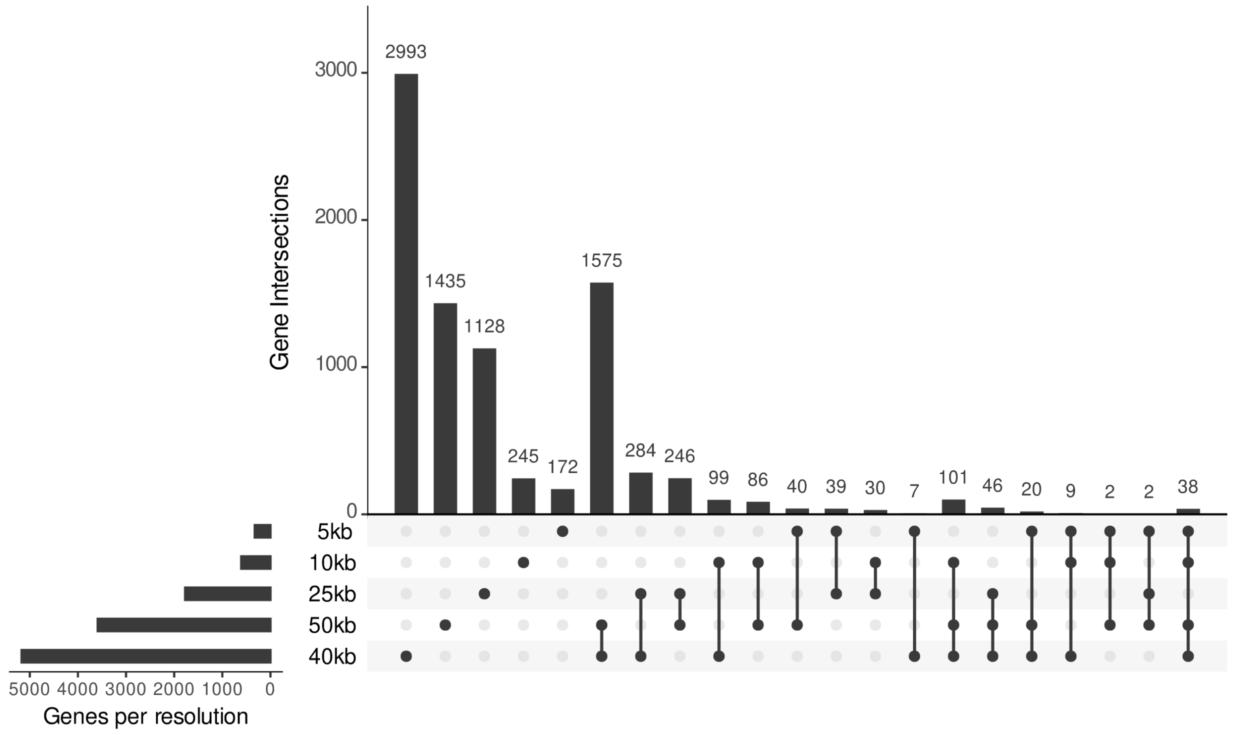

Figure S13. Upset plot showing intersections among genes in most similar regions between HFFc6 and H1-hESC identified for different contact matrix resolutions (see Supplementary Fig. S12 for details).

Supplementary Tables

Table S1. Hi-C data sets. BR: biological replicate.

Cell line	BR	`BAM` Accession
G401	1	ENCFF649MAY
G401	2	ENCFF758WUD
LNCaP	1	ENCFF977XHB
LNCaP	2	ENCFF204XII
A549	1	ENCFF867DCM
A549	2	ENCFF532XBC

Table S2. Micro-C data sets. BR: biological replicate.

Cell line	BR	`pairs` Accession
H1-hESC	1	4DNFING6ZFD, 4DNFIBMG8YA3, 4DNFIMT4PHZ1, 4DNFI8GM4EL9
H1-hESC	2	4DNFIIYUGYBU, 4DNFI89L17XY, 4DNFIXP9MVBU, 4DNFI2YHYAJO, 4DNFIULY29IQ
HFFc6	1	4DNFIN7IIIY6, 4DNFIJZDEIZ3, 4DNFIYBTHGNA, 4DNFIK8UIB5B
HFFc6	2	4DNFIF5F4HRG, 4DNFIK82YRNM, 4DNFIATCW955, 4DNFIZU6ADT1, 4DNFIKWV6BY2
HFFc6	3	4DNFIFJL4JIH, 4DNFIONHB78N, 4DNFIG1ZOVIM, 4DNFIPKVL9YI, 4DNFIJM966UR, 4DNFIV8JNJB8

Table S3. Number of interactions in each sample. BR: biological replicate.

Cell line	BR1	BR2	BR3
H1-hESC	1,255,082,197	153,084,436	
HFFc6	2,857,647,629	830,616,793	1,423,953,776
G401	100,949,135	101,518,640	
LNCaP	53,446,400	35,777,493	
A549	49,612,977	47,475,253	

Table S4. Downsampling of the analyzed data sets. 1 indicates that the data set was downsampled to ”Nr. Interactions” and 0 that it was not.

Nr. Interactions	H1-hESC	HFFc6	G401	LNCaP	A549
1 million	1	1	1	1	1
5 million	1	1	1	1	1
10 million	1	1	1	1	1
20 million	1	1	1	1	1
25 million	1	1	1	1	1
30 million	1	1	1	1	1
60 million	1	1	1	0	0
120 million	1	1	0	0	0
240 million	1	1	0	0	0
400 million	1	1	0	0	0
800 million	1	1	0	0	0
